# Bevacizumab and gamma knife radiosurgery for first-recurrence glioblastoma

**DOI:** 10.1007/s11060-023-04524-y

**Published:** 2024-01-04

**Authors:** Jeff F. Zhang, Bernard Okai, Austin Iovoli, Victor Goulenko, Kristopher Attwood, Jaims Lim, Ryan M. Hess, Ajay P. Abad, Dheerendra Prasad, Robert A. Fenstermaker

**Affiliations:** 1 Jacobs School of Medicine and Biomedical Sciences, State University of New York at Buffalo, Buffalo, NY USA; 2 Department of Radiation Medicine, Roswell Park Comprehensive Cancer Center, Buffalo, NY USA; 3 Department of Biostatistics and Bioinformatics, Roswell Park Comprehensive Cancer Center, Buffalo, NY USA; 4 Department of Neurosurgery, Jacobs School of Medicine and Biomedical Sciences, State University of New York at Buffalo, Buffalo, NY USA; 5 Department of Neuro-Oncology, Roswell Park Comprehensive Cancer Center, Buffalo, NY USA; 6 Department of Neurosurgery, Roswell Park Comprehensive Cancer Center, Elm and Carlton Streets, Buffalo, NY 14263 USA

**Keywords:** Bevacizumab, CNS tumor, Gamma Knife, Glioblastoma, Stereotactic radiosurgery, Temozolomide

## Abstract

**Introduction:**

Glioblastoma (GBM) is the most common central nervous system malignancy in adults. Despite decades of developments in surgical management, radiation treatment, chemotherapy, and tumor treating field therapy, GBM remains an ultimately fatal disease. There is currently no definitive standard of care for patients with recurrent glioblastoma (rGBM) following failure of initial management.

**Objective:**

In this retrospective cohort study, we set out to examine the relative effects of bevacizumab and Gamma Knife radiosurgery on progression-free survival (PFS) and overall survival (OS) in patients with GBM at first-recurrence.

**Methods:**

We conducted a retrospective review of all patients with rGBM who underwent treatment with bevacizumab and/or Gamma Knife radiosurgery at Roswell Park Comprehensive Cancer Center between 2012 and 2022. Mean PFS and OS were determined for each of our three treatment groups: Bevacizumab Only, Bevacizumab Plus Gamma Knife, and Gamma Knife Only.

**Results:**

Patients in the combined treatment group demonstrated longer post-recurrence median PFS (7.7 months) and median OS (11.5 months) compared to glioblastoma patients previously reported in the literature, and showed improvements in total PFS (p=0.015), total OS (p=0.0050), post-recurrence PFS (p=0.018), and post-recurrence OS (p=0.0082) compared to patients who received either bevacizumab or Gamma Knife as monotherapy.

**Conclusion:**

This study demonstrates that the combined use of bevacizumab with concurrent stereotactic radiosurgery can have improve survival in patients with rGBM.

**Supplementary Information:**

The online version contains supplementary material available at 10.1007/s11060-023-04524-y.

## Introduction

Glioblastoma (GBM) is the most common malignant brain cancer in adults and accounts for 25–30% of all primary central nervous system (CNS) tumors [1]. Despite developments in various treatment modalities over the past three decades, the prognosis for extended survival remains poor, with various studies showing median progression-free survival (PFS) and overall survival (OS) ranging from 6.2–7.5 months and 14.6–16.7 months, respectively, from time of initial diagnosis [2]. Standard-of-care treatment of GBM commonly consists of maximal safe surgical resection and radiotherapy followed by chemotherapy with temozolomide [3]. Other forms of treatment including tumor treating fields, carmustine wafers, and bevacizumab are also commonly used as adjuvants to primary therapy [4, 5]. However, tumor recurrence usually develops within 6 months following initial treatment [6, 7], most often within 2 cm of the surgical cavity [1, 8].

There are currently no established standard therapies for recurrent GBM consistently showing improvements in OS. Joint recommendations from the American Association of Neurological Surgeons and Congress of Neurological Surgeons support re-resection (Level II) [9], re-irradiation (Level III) [10], and revised temozolomide dosing (Level III) [11]. The use of nitrosoureas, platinum agents, topoisomerase inhibitors, tumor treating field therapy, and viral therapy are either not recommended or have insufficient evidence for recommendation (Level III) [11]. Bevacizumab has been shown to improve PFS in this setting, but there is currently insufficient evidence to show advantages in PFS or OS when it is used in combination with cytotoxic chemotherapeutic agents (Level III) [12]. Despite aggressive treatment, patients with rGBM have an estimated median PFS of 9 weeks and median OS of 25 weeks following tumor recurrence [13].

We performed a single-institution, retrospective analysis of all patients treated for rGBM over a 10-year period between 2012 and 2022. Treatment of these first-recurrence GBM patients (who failed initial Stupp protocol management) included administration of bevacizumab and/or treatment with Gamma Knife radiosurgery.

## Methods

Following Institutional Review Board (IRB) approval, we conducted a retrospective review of all patients diagnosed with recurrent GBM who were treated with bevacizumab and/or Gamma Knife radiosurgery at the Roswell Park Comprehensive Cancer Center between September 1, 2012, and April 30, 2022. All diagnoses of GBM were made upon histopathological analysis of tissue samples obtained during biopsy or surgical resection, consistent with World Health Organization diagnostic criteria at the time of tissue resection [14, 15]. Patients were retrospectively assigned to one of three treatment groups depending upon whether they had received bevacizumab monotherapy (“BEV Only”), Gamma Knife monotherapy (“GK Only”), or a combination of the two (“BEV + GK”). Patients were excluded from further data collection and analysis if they had incomplete records or no available post-treatment magnetic resonance imaging (MRI), were participating in a clinical trial, did not complete Stupp protocol treatment, or had evidence of leptomeningeal disease at the time of diagnosis or recurrence. Patients who were reported to be alive at the time of data collection were also excluded due to the inability to definitively calculate PFS and OS for these patients.

Gamma Knife stereotactic radiosurgery (SRS) was performed using the Leksell Gamma Knife Perfexion and Icon (Elekta Inc) systems. Treatment planning was carried out using pretreatment MRIs imported into Leksell Gamma Plan (Version 11.0.2) and dosing (ranging from 0–30 Gy in 1–5 fractions) was calculated for each patient specifically based on tumor size and location. No margins were added to the gross tumor volume in order to determine the clinical or planned tumor volumes. Tumor volumes were calculated from manual segmentation of pre-treatment and post-treatment MRIs using Brainlab (Version 1.6.2.54) and Eclipse External Beam Planning (Varian, Version 15.6) software programs.

Data analysis was carried out using Microsoft Excel (Microsoft Office, Version 16.0.5278.1000) and IBM SPSS Statistics (Version 29.0.0.0). Patient demographic and clinical variables were compared across treatment groups using one-way analysis of variance (ANOVA), Mann–Whitney, Kruskal–Wallis, and chi-square testing. Univariate and multivariate Cox proportional hazards regression models were used to evaluate the association between survival outcomes and treatment groups while accounting for potential confounders: age, gender, single vs. multifocal lesions, recurrence tumor volume, IDH and MGMT statuses, post-surgical KPS, number of bevacizumab cycles, steroid use, number of surgical resections, and number of other chemotherapeutic agents administered to the patients during their treatment course.

## Results

### Patient Demographics

Two-hundred and forty-eight patients received treatment for rGBM at the Roswell Park Comprehensive Cancer Center between September 1, 2012, and April 30, 2022. Of those patients, 153 were excluded for the following reasons: 90 patients had incomplete treatment records, 24 patients were alive at the time of data collection, 17 patients were participants in a clinical trial, 15 patients had refused or been unable to complete standard of care management, five patients had diffuse leptomeningeal disease at the time of diagnosis, one patient had received more than three craniotomies in the past for treatment of astrocytoma, and one patient had been receiving chronic bevacizumab for low-grade glioma prior to starting treatment for GBM.

Patient demographics and tumor characteristics are presented in Table 1. Of the 95 rGBM patients included in our study, 19 patients were treated with bevacizumab only (BEV Only), 57 patients were treated with both bevacizumab and Gamma Knife (BEV + GK), and 19 patients received Gamma Knife SRS only (GK Only). The mean age at diagnosis was 56.8 ± 11.3 years, with no significant differences in patient ages among treatment groups (p = 0.23). The median number of surgical resections was higher in the BEV + GK group compared to BEV Only and GK Only patients (2 vs. 1 vs. 1, p = 0.012, respectively). The number of bevacizumab cycles received by patients in the BEV Only and BEV + GK groups did not significantly differ (BEV Only vs. BEV + GK: 5 vs. 5, p = 0.27), and the use of steroids (p = 0.34) and number of other chemotherapeutic agents received during treatment for rGBM (p = 0.28) were also similar across all treatment groups. The median decline in KPS from post-surgical functioning to the last recorded KPS found in each patient’s note was 20 for all patients, with no significantly increased or decreased declines noted for any particular treatment group (p = 0.79).

**Table 1:** Patient Demographics and Clinical Variables

	Population (n = 95)	BEV Only (n = 19)	BEV + GK (n = 57)	GK Only (n = 19)	p-value
Age (mean ± SD) (years)	56.8 ± 11.3	59.8 ± 12.1	56.8 ± 9.6	53.5 ± 14.4	0.23
Male (n, %)	61 (64.2)	12 (63.2)	36 (63.2)	13 (68.4)	0.91
Female (n, %)	34 (35.8)	7 (36.8)	21 (36.8)	6 (31.6)	0.91
Number of Surgical Resections (median, IQR)	1 (1–2)	1 (1–1)	2 (1–2)	1 (1–2)	0.012
Steroid Use (n, %)	87 (91.6)	17 (89.5)	54 (94.7)	16 (84.2)	0.34
Number of BEV Cycles (median, IQR)	5 (3.5–8)	5 (2–6)	5 (4–9.5)	0 (0–0)	0.27
Number of Other Chemotherapeutic Agents (median, IQR)	1 (1–2)	1 (0–1)	1 (1–2)	1 (0–2)	0.28
Post-Surgical KPS (median, IQR)	70 (70–80)	70 (60–80)	80 (70–90)	70 (60–80)	0.12
Last Recorded KPS (median, IQR)	50 (40–60)	50 (40–60)	50 (40–60)	60 (40–70)	0.89
KPS Decline (median, IQR)	20 (10–30)	30 (0–30)	20 (10–40)	20 (0–30)	0.79
GBM Location					
Single Lesion (n, %)	54 (56.8)	12 (63.2)	34 (59.6)	8 (42.1)	0.34
Multifocal Lesion (n, %)	41 (43.2)	7 (36.8)	23 (40.4)	11 (57.9)	0.34
Left-Sided (n, %)	45 (47.4)	11 (57.9)	30 (52.6)	4 (21.1)	0.034
Right-Sided (n, %)	53 (55.8)	9 (47.4)	29 (50.9)	15 (78.9)	0.073
Bifrontal (n, %)	1 (1.1)	1 (5.3)	0 (0.0)	0 (0.0)	0.13
Temporal (n, %)	41 (43.2)	6 (31.6)	25 (43.9)	10 (52.6)	0.42
Frontal (n, %)	33 (34.7)	6 (31.6)	21 (36.8)	6 (31.6)	0.87
Parietal (n, %)	22 (23.2)	5 (26.3)	13 (22.8)	4 (21.1)	0.92
Occipital (n, %)	4 (4.2)	1 (5.3)	2 (3.5)	1 (5.3)	0.92
Cerebellum (n, %)	2 (2.1)	1 (5.3)	0 (0.0)	1 (5.3)	0.22
Interventricular (n, %)	1 (1.1)	0 (0.0)	1 (1.8)	0 (0.0)	0.71
Corpus Callosum (n, %)	1 (1.1)	0 (0.0)	0 (0.0)	1 (5.3)	0.13
Insula (n, %)	1 (1.1)	1 (5.3)	0 (0.0)	0 (0.0)	0.13
Thalamus (n, %)	1 (1.1)	0 (0.0)	1 (1.8)	0 (0.0)	0.71
Pons (n, %)	1 (1.1)	1 (5.3)	0 (0.0)	0 (0.0)	0.13
Initial Tumor Volume (cm^3^) (mean ± SD)	28.8 ± 24.3	21.5 ± 21.9	30.2 ± 22.9	32.2 ± 30.1	0.32
Post-Surgical Tumor Volume (cm^3^) (mean ± SD)	3.2 ± 3.3	3.3 ± 3.7	2.8 ± 3.1	4.3 ± 3.3	0.021
Recurrence Tumor Volume (cm^3^) (mean ± SD)	10.4 ± 13.0	15.9 ± 11.5	7.6 ± 11.6	13.2 ± 16.1	0.030
First Post-Treatment Tumor Volume (cm^3^) (mean ± SD)	6.8 ± 8.4	7.9 ± 8.0	5.0 ± 5.7	10.9 ± 13.1	0.024
Second Post-Treatment Tumor Volume (cm^3^) (mean ± SD)	7.7 ± 11.2	8.2 ± 7.6	7.7 ± 12.2	5.2 ± 6.6	0.91
Third Post-Treatment Tumor Volume (cm^3^) (mean ± SD)	8.5 ± 12.1	11.1 ± 11.1	7.9 ± 12.3	-	0.43
Last Recorded Tumor Volume (cm^3^) (mean ± SD)	17.6 ± 19.7	13.0 ± 10.3	20.9 ± 23.3	12.6 ± 12.0	0.15
IDH-WT, MGMT-Unmethylated (n, %)	49 (69.0)	13 (81.3)	32 (69.6)	4 (44.4)	0.16
IDH-WT, MGMT-Methylated (n, %)	16 (22.5)	1 (6.3)	12 (26.1)	3 (33.3)	0.19
IDH-Mutated, MGMT-Unmethylated (n, %)	4 (5.6)	1 (6.3)	1 (2.2)	2 (22.2)	0.058
IDH-Mutated, MGMT-Methylated (n, %)	2 (2.8)	1 (6.3)	1 (2.2)	0 (0.0)	0.60

Age is reported as patient age at first diagnosis of glioblastoma. KPS Decline was determined by taking the difference of each patient’s Post-Surgical KPS and Last Recorded KPS. Patient stratification and analysis of subgroup sizes and proportions based on IDH and MGMT statuses were calculated only for the n = 71 patients for whom both IDH and MGMT statuses were available. p-values were calculated between the “BEV Only,” “BEV + GK,” and “GK Only” patient subgroups. Mann–Whitney U-testing was used to compare medians for discrete variables; ANOVA testing was used to compare means for continuous variables

*BEV* bevacizumab; *GBM* glioblastoma; *GK* Gamma Knife; *IDH* isocitrate dehydrogenase; *IQR* interquartile range; *KPS* Karnofsky performance scale; *MGMT* O [6]-methylguanine-DNA methyltransferase; *SD* standard deviation; *WT* wild-type

The majority of GBMs were right-hemispheric (n = 53, 55.8%). Three patients had multifocal lesions involving both left and right hemispheres. Single focus lesions were present in 56.8% (n = 54) of study patients, while 43.2% (n = 41) of patients had multifocal lesions. The majority of lesions occurred in the temporal lobe (n = 41, 43.2%), followed by the frontal lobe (n = 33, 34.7%), parietal lobe (n = 22, 23.2%), and occipital lobe (n = 4, 4.2%). Five tumors involved the lateral ventricle, corpus callosum, insular cortex, thalamus, and pons. There were no significant differences in the distribution of GBM locations among the three treatment groups.

The mean initial tumor and post-surgical volumes were 28.8 ± 24.3 cm^3^ and 3.2 ± 3.3 cm^3^. Patients in the BEV + GK treatment group had the lowest mean post-surgical, recurrence, and first post-treatment tumor volumes (p = 0.021, 0.030, and 0.024, respectively), but no significant differences in second, third, or last recorded tumor volumes were noted. In total, 213 target lesions were treated with Gamma Knife radiosurgery. No patients received a third GK treatment, and statistical testing for differences in “Third Post-Treatment Tumor Volumes” was therefore only performed between the BEV Only and BEV + GK groups.

Only 71 of 95 (74.7%) included patients had both IDH and MGMT statuses available. Forty-nine patients (69.0%) were IDH-wild type, MGMT-unmethylated; 16 patients (22.5%) were IDH-wild type, MGMT-methylated; four patients (5.6%) were IDH-mutated, MGMT-unmethylated; and two patients (2.8%) were IDH-mutated, MGMT-methylated. No significant differences in the distribution of these patients throughout the three treatment groups were found.

### Univariate and Multivariate Survival Outcomes

The median PFS and OS for our study population were 14.1 months and 17.1 months, respectively. Patients in the BEV + GK group had the highest median PFS (BEV Only vs. BEV + GK vs. GK Only: 11.8 vs. 15.6 vs. 12.0 months, p = 0.015) and OS (BEV Only vs. BEV + GK vs. GK Only: 14.3 vs. 18.6 vs. 15.0 months, p = 0.0050) among treatment groups. Patients in the combination treatment group also showed significantly improved post-tumor recurrence PFS and OS compared to patients in the individual treatment groups (Post-Recurrence PFS, BEV Only vs. BEV + GK vs. GK Only: 5.0 vs. 7.7 vs. 4.9 months, p = 0.018; Post-Recurrence OS, BEV Only vs. BEV + GK vs. GK Only: 6.5 vs. 11.5 vs. 7.9 months, p = 0.0082).

Patients were also stratified by IDH and MGMT statuses vs. treatment group, with the highest PFS and OS found for the “IDH-WT, MGMT-Methylated” genotype (PFS: 17.0 months, OS: 21.2 months) among all patients included in this study. Patients with “IDH-WT, MGMT-Unmethylated” did not show differences in PFS or OS when stratified by treatment group, and statistical analysis of patients with other IDH/MGMT combination genotypes were not able to be performed due to small subgroup sizes. These results are presented in Table 2.

**Table 2:** Patient Survival Outcomes

	Population (n = 95)	BEV Only (n = 19)	BEV + GK (n = 57)	GK Only (n = 19)	p-value
PFS (median, CI) (months)	14.1 (15.2–20.0)	11.8 (9.6–14.8)	15.6 (16.2–23.1)	12.0 (10.7–22.3)	0.015
OS (median, CI) (months)	17.1 (18.7–24.0)	14.3 (11.6–17.9)	18.6 (20.3–27.8)	15.0 (13.4–25.2)	0.0050
Post-Recurrence PFS (median, CI) (months)	6.1 (6.9–9.2)	5.0 (4.1–6.8)	7.7 (7.5–10.7)	4.9 (4.7–10.0)	0.018
Post-Recurrence OS (median, CI) (months)	10.0 (10.2–13.4)	6.5 (6.3–10.0)	11.5 (11.3–15.8)	7.9 (7.0–13.2)	0.0082
PFS (median, CI) (months)					
IDH-WT, MGMT-Unmethylated	14.1 (14.2–21.5)	12.1 (9.2–16.0)	15.6 (14.5–24.8)	15.1 (0.0–41.6)	0.55
IDH-WT, MGMT-Methylated	17.0 (14.8–26.7)	–	–	–	–
IDH-Mutated, MGMT- Unmethylated	8.2 (−21.6–54.5)	–	–	–	–
IDH-Mutated, MGMT-Methylated	10.8 (−28.4–49.9)	–	–	–	–
OS (median, CI) (months)					
IDH-WT, MGMT-Unmethylated	16.7 (17.4–25.2)	14.6 (10.9–19.2)	17.8 (18.0–28.7)	18.5 (0.46–49.1)	0.58
IDH-WT, MGMT-Methylated	21.2 (18.6–32.9)	–	–	–	–
IDH-Mutated, MGMT- Unmethylated	9.3 (−24.0–61.8)	–	–	–	–
IDH-Mutated, MGMT-Methylated	(−27.7–54.9)	–	–	–	–

PFS and post-recurrence PFS durations were calculated from date of initial diagnosis or date of tumor recurrence to date of post-treatment progression, respectively. OS and post-recurrence OS durations were calculated from date of initial diagnosis or date of tumor recurrence to date of patient death. PFS and OS durations were stratified by IDH and MGMT status subgroups for the n = 71 patients for whom both IDH and MGMT statuses were available. Statistical analysis was not suitable for the “IDH-Mutated, MGMT-Unmethylated” and “IDH-Mutated, MGMT-Methylated” subgroups due to small subgroup sizes (n = 4 and n = 2, respectively). Survival results were presented as “median (95% confidence interval).” p-values were calculated between the “BEV Only,” “BEV + GK,” and “GK Only” patient subgroups using Kruskal–Wallis testing to compare median values

*BEV*, bevacizumab; *CI* confidence interval; *GK* Gamma Knife; *IDH* isocitrate dehydrogenase; *MGMT* O [6]-methylguanine-DNA methyltransferase; *OS* overall survival; *PFS* progression-free survival; *SD* standard deviation; *WT* wild-type

**Fig. 1:**
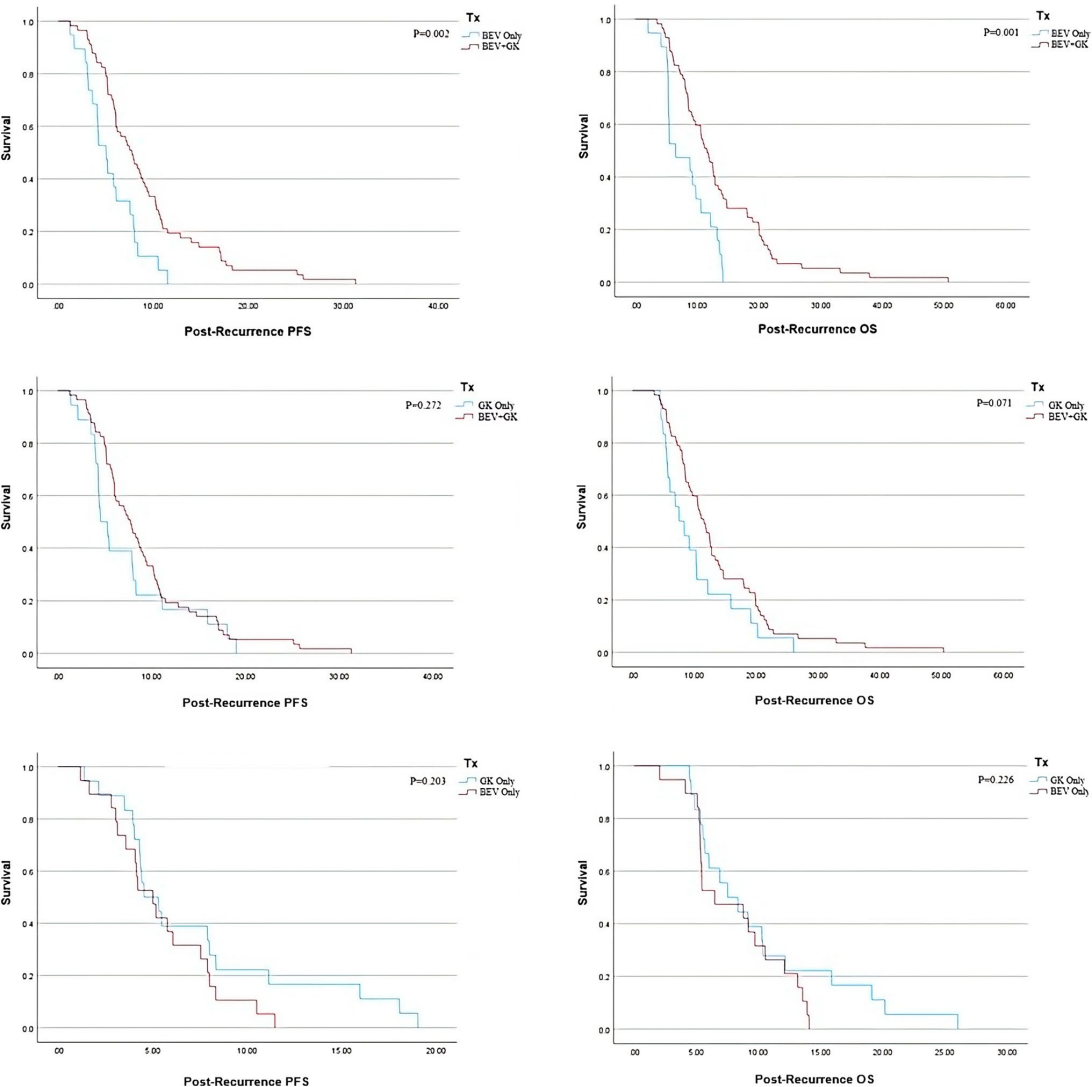
Post-Recurrence Survival Plots. Kaplan–Meier survival plots as a function of post-recurrence PFS or OS compared between treatment groups. The x-axis represents post-recurrence PFS or OS durations in months. The y-axis represents the cumulative survival probability. *BEV* bevacizumab; *GK* Gamma Knife; *OS* overall survival; *PFS* progression-free survival; *Tx* treatment

Kaplan–Meier plots (Fig. 1) were constructed to compare cumulative survival functions for post-recurrence PFS and OS between the three treatment groups. Only the comparison between BEV Only and BEV + GK groups showed significant differences in post-recurrence PFS (p = 0.002) and OS (p = 0.001).

Univariate regression analysis was performed for PFS, OS, post-recurrence PFS, and post-recurrence OS with respect to a number of our study demographic and clinical variables, including age, gender, GBM location, single vs. multifocal lesion, IDH and MGMT statuses, tumor volumes at various timepoints, administered medications, number of surgical resections, and KPS (Table 3). None of the tested variables were associated with consistent increases or decreases in risk of disease progression or patient death. However, patient age was associated with significantly increased risk of early progression (PFS: HR = 1.07, p = 0.003) and patient death (OS: HR = 1.05, p = 0.009; Post-Recurrence OS: HR = 1.05, p = 0.025) for three of our four survival metrics, while a greater number of surgical resections was associated with significantly decreased risk of progression (PFS: HR = 0.44, p = 0.002; Post-Recurrence PFS: HR = 0.46, p = 0.015) and patient death (OS: HR = 0.40, p < 0.001) for three of our four survival metrics.

**Table 3:** Univariate Survival Outcomes by Demographic and Clinical Variables

Variable	PFS	OS	Post-Recurrence PFS	Post-Recurrence OS
Age	1.07 (1.02–1.12) p = 0.003	1.05 (1.01, 1.09) p = 0.009	1.04 (1.00–1.08), p = 0.072	1.05 (1.01–1.09), p = 0.025
Gender: Male	1.67 (0.77–3.61), p = 0.19	1.56 (0.76–3.23), p = 0.23	1.08 (0.49–2.37), p = 0.86	1.47 (0.70–3.10), p = 0.31
GBM Location: Bifrontal	18.00 (0.18–1791.88), p = 0.22	3.45 (0.04–274.74), p = 0.58	0.12 (0.00–5.99), p = 0.29	0.36 (0.01–15.42), p = 0.60
GBM Location: Left Frontal	1.04 (0.22–5.02), p = 0.96	0.65 (0.12–3.45), p = 0.62	0.06 (0.01–0.50), p = 0.009	0.15 (0.03–0.88), p = 0.035
GBM Location: Left Insular	1.41 (0.11–18.74), p = 0.79	0.72 (0.05–10.35), p = 0.81	0.01 (0.00–0.18), p = 0.002	0.04 (0.00–0.54), p = 0.02
GBM Location: Left Interventricular	0.40 (0.03–5.44), p = 0.49	0.14 (0.01–2.20), p = 0.16	0.01 (0.00–0.10), p < 0.001	0.02 (0.00–0.26), p = 0.003
GBM Location: Left Occipital	0.12 (0.01–2.20), p = 0.15	0.14 (0.01–2.38), p = 0.17	0.04 (0.00–1.07), p = 0.055	0.18 (0.01–3.00), p = 0.24
GBM Location: Left Parietal	2.78 (0.47–16.50), p = 0.26	2.81 (0.46–17.23), p = 0.27	0.20 (0.03–1.15), p = 0.071	0.78 (0.15–3.98), p = 0.77
GBM Location: Left Temporal	1.86 (0.33–10.53), p = 0.49	0.84 (0.12–5.75), p = 0.86	0.10 (0.02–0.65), p = 0.016	0.26 (0.05–1.46), p = 0.13
GBM Location: Right Frontal	0.55 (0.12–2.49), p = 0.44	0.40 (0.08–2.06), p = 0.27	0.32 (0.07–1.50), p = 0.15	0.50 (0.12–2.11), p = 0.35
GBM Location: Right Parietal	0.42 (0.06–2.73), p = 0.36	0.16 (0.02–1.27), p = 0.083	0.10 (0.02–0.64), p = 0.015	0.08 (0.01–0.46), p = 0.005
GBM Location: Right Pons	20.11 (1.24–326.74), p = 0.035	17.98 (0.96–337.95), p = 0.054	0.10 (0.01–1.36), p = 0.084	0.27 (0.02–3.22), p = 0.30
GBM Location: Right Temporal	2.16 (0.55–8.54), p = 0.27	1.68 (0.39–7.32), p = 0.49	0.79 (0.16–3.93), p = 0.78	0.98 (0.19–5.09), p = 0.98
Lesion: Single-Focus	1.46 (0.56–3.82), p = 0.44	2.07 (0.78–5.47), p = 0.14	5.15 (1.67–15.88), p = 0.004	2.57 (1.02–6.43), p = 0.045
IDH Status: Mutated	0.36 (0.04–3.60), p = 0.39	0.39 (0.04–3.79), p = 0.42	0.70 (0.10–4.91), p = 0.72	0.36 (0.05–2.67), p = 0.32
MGMT Status: Methylated	0.77 (0.29–2.04), p = 0.60	0.46 (0.17–1.27), p = 0.14	0.42 (0.13–1.40), p = 0.16	0.38 (0.12–1.23), p = 0.11
Initial Tumor Volume	0.99 (0.97–1.01), p = 0.18	0.99 (0.97–1.01), p = 0.23	0.99 (0.97–1.00), p = 0.11	0.99 (0.97–1.01), p = 0.17
Post-Surgical Tumor Volume	1.10 (0.96–1.27), p = 0.18	1.12 (0.97–1.29), p = 0.11	1.07 (0.92–1.23), p = 0.38	1.07 (0.94–1.21), p = 0.29
Recurrence Tumor Volume	0.98 (0.93–1.03), p = 0.42	0.99 (0.95–1.04), p = 0.75	0.99 (0.96–1.03), p = 0.70	1.00 (0.97–1.04), p = 0.87
Last Recorded Tumor Volume	1.02 (0.99–1.04), p = 0.30	1.02 (0.99–1.04), p = 0.18	1.00 (0.97–1.03), p = 0.78	1.01 (0.99–1.04), p = 0.28
Number of BEV Cycles	0.97 (0.92–1.03), p = 0.31	0.96 (0.90–1.02), p = 0.15	0.78 (0.67–0.90), p = 0.001	0.86 (0.78–0.95), p = 0.003
Number of Other Chemotherapeutic Agents	1.37 (0.94–2.01), p = 0.11	1.24 (0.88–1.76), p = 0.22	0.97 (0.62–1.51), p = 0.89	0.76 (0.53–1.10), p = 0.15
Steroid Use	0.44 (0.07–2.77), p = 0.39	1.58 (0.27–9.19), p = 0.61	1.16 (0.20–6.87), p = 0.87	5.78 (0.82–40.74), p = 0.078
Number of Surgical Resections	0.44 (0.26–0.75), p = 0.002	0.40 (0.24–0.67), p < 0.001	0.46 (0.24–0.86), p = 0.015	0.61 (0.34–1.09), p = 0.092
Post-Surgical KPS	1.00 (0.97–1.02), p = 0.82	1.00 (0.97–1.02), p = 0.90	1.00 (0.97–1.02), p = 0.71	0.98 (0.96–1.01), p = 0.20

Hazard Ratios with 95% confidence intervals and associated p-values were constructed between the Variable and the survival metric (PFS, OS, Post-Recurrence PFS, or Post-Recurrence OS)

*BEV* Bevacizumab; *GBM* glioblastoma; *IDH* isocitrate dehydrogenase; *KPS* Karnofsky performance scale; *MGMT* O [6]-methylguanine-DNA methyltransferase; *OS* overall survival; *PFS* progression-free survival

Cox multivariate regression models (Table 4) were constructed to compare survival outcomes between the BEV Only, BEV + GK, and GK Only treatment groups while controlling for intergroup differences in age, gender, single vs. multifocal lesion, recurrence tumor volume, IDH and MGMT statuses, post-surgical KPS, number of bevacizumab cycles, steroid use, number of surgical resections, and number of other chemotherapeutic agents administered to the patients during their treatment course. Patients in the combination treatment group showed significant benefits in post-recurrence PFS and OS in comparison to the BEV Only group (BEV + GK vs. BEV Only, Post-Recurrence PFS: HR = 0.44, p = 0.028; BEV + GK vs. BEV Only, Post-Recurrence OS: HR = 0.32, p = 0.004), but not in comparison to the GK Only group (BEV + GK vs. GK Only, Post-Recurrence PFS: HR = 1.45, p = 0.37; BEV + GK vs. GK Only, Post-Recurrence OS: HR = 1.27, p = 0.58). The GK Only also showed significant improvements in Post-Recurrence PFS and OS in comparison to the BEV Only group on multivariate analysis (BEV Only vs. GK Only, Post-Recurrence PFS: HR = 8.19, p = 0.0080; BEV Only vs. GK Only, Post-Recurrence OS: HR = 5.58, p = 0.032).

**Table 4:** Multiple Cox Regression Models for PFS and OS by Treatment Group

Covariate	Hazard Ratio	p-value
PFS: BEV + GK vs. BEV Only	0.63 (0.28–1.43)	0.27
PFS: BEV + GK vs. GK Only	0.80 (0.35–1.84)	0.60
PFS: BEV Only vs. GK Only	0.86 (0.12–6.00)	0.88
Post-Recurrence PFS: BEV + GK vs. BEV Only	0.44 (0.21–0.92)	0.028
Post-Recurrence PFS: BEV + GK vs. GK Only	1.45 (0.64–3.28)	0.37
Post-Recurrence PFS: BEV Only vs. GK Only	8.19 (1.71–39.14)	0.0080
OS: BEV + GK vs. BEV Only	0.63 (0.27–1.50)	0.30
OS: BEV + GK vs. GK Only	0.73 (0.32–1.66)	0.45
OS: BEV Only vs. GK Only	0.94 (0.16–5.65)	0.95
Post-Recurrence OS: BEV + GK vs. BEV Only	0.32 (0.15–0.70)	0.0040
Post-Recurrence OS: BEV + GK vs. GK Only	1.27 (0.55–2.92)	0.58
Post-Recurrence OS: BEV Only vs. GK Only	5.58 (1.16–26.80)	0.032

Hazard Ratios with 95% confidence intervals and associated p-values were constructed between the covariates and their effect on the survival metric (PFS, OS, Post-Recurrence PFS, or Post-Recurrence OS)

*BEV* bevacizumab; *GK* gamma knife; *OS* overall survival; *PFS* progression-free survival

## Discussion

Few studies have examined the combined use of bevacizumab and stereotactic radiosurgery in the treatment of recurrent GBM. Case series suggest promising but inconclusive results due to their small sample sizes and insufficient controls for demographic and treatment variability [16, 17]. A recent study by Morris et al. reported improvements in PFS and OS in rGBM patients treated with bevacizumab and Gamma Knife versus Gamma Knife alone when combining their results with other published studies [18]. However, a formal meta-analysis was not performed to reach this conclusion and significant variability existed between the study populations, as was stated by the authors. In our study, we examined whether bevacizumab and Gamma Knife SRS together, or as separate treatments, result in improved survival in rGBM by accounting for intergroup differences in patient demographics, tumor characteristics, number of surgical resections, functional scores, and other concurrently administered steroid and chemotherapeutic medications. Our results showed that patients treated with both bevacizumab and Gamma Knife had improved total and post-recurrence PFS and OS compared to patients previously reported in the literature [2], and was associated with superior survival outcomes compared to patients treated with bevacizumab or Gamma Knife alone.

Glioblastoma cells have been shown to produce high levels of VEGF which support angiogenesis, neovascularization, and tumor growth. In addition to being associated with higher recurrence rates and poorer patient prognosis [19, 20], increased levels of VEGF signaling promote the proliferation and tumorigenic properties of glioma stem cells, which are highly resistant to chemotherapy and radiation [21, 22]. Therefore, anti-VEGF agents such as bevacizumab could be included in standard treatment regimens for GBM in order to inhibit angiogenesis and slow tumor growth, while also controlling the development of abnormal peritumoral blood vessels to increase the delivery of systemic chemotherapeutic drugs [23–25]. Bevacizumab has been utilized as an antiangiogenic agent in the treatment of other forms of cancer and its use is associated with significant improvements in OS and PFS in advanced colorectal cancer [26], ovarian cancer [27], non-small cell lung cancer [28], and cervical cancer [29]. The effectiveness of bevacizumab in the treatment of GBM, on the other hand, is more controversial, and major Phase III trials have previously only shown improvements in PFS without any effects on OS [30–32]. However, a recent Cochrane review which included 11 randomized, controlled trials studying the use of antiangiogenic agents in the treatment of both newly-diagnosed and recurrent high-grade glioma, found a PFS benefit as well as modest improvements in OS for both disease groups when antiangiogenic agents were given in combination with chemotherapy (reported as HR = 0.92, p = 0.05) [33].

The mechanism by which radiation treatment controls tumor growth is complex, but is thought to include to the production of free radicals which damage DNA and trigger apoptotic signaling pathways in proliferating cells [1]. SRS treatment also alters the microvasculature of tumors, resulting in decreased capillary density and stenosis of blood vessels in the affected area [34]. The efficacy of SRS is therefore in part related to the radiosensitivity of endothelial cells which comprise the tumor vasculature [35]. Tumor cells are capable of developing radioresistance through the production of cytokines that have protective and proliferative effects on endothelial cells [36]. In particular, radiation exposure has been shown to greatly increase the expression of HIF-1 with downstream induction of VEGF production by tumor cells, triggering the formation of new peritumoral vasculature to replace damaged and narrowed vessels [36]. Therefore, mechanistically, we thought pairing bevacizumab with SRS would constitute a logical therapeutic paradigm. A meta-analysis by Larson et al. of 9 studies from 2005 to 2013 found that median OS in rGBM was significantly higher (range 16.7 to 33.3 months) in the group treated with adjunct Gamma Knife radiosurgery, with the greatest survival benefit observed in the subgroup of patients who received re-resection or bevacizumab along with Gamma Knife [6].

Bevacizumab has previously been used as an adjunct to radiation treatment in order to ameliorate symptoms caused by cerebral edema through the “normalization” of weak, highly permeable vessels in the irradiated area. This hypothetically would allow for higher and better tolerated radiation doses, which has been suggested could improve PFS and OS [16, 20, 37]. Our study presents the largest sample population to date of patients treated with this combined regimen for rGBM. Patients in the BEV + GK and GK Only groups showed improved PFS and OS compared to other GBM patients reported in the literature, with significant improvements in survival outcome associated with the combination treatment group. Consistent with previous reports, we also found that patients who received only bevacizumab showed improvements in PFS but not in OS.

While post-recurrence survival outcomes in the GK Only group showed significant improvements over the BEV Only group on multivariate analysis, our population results showed that maximal survival benefits were observed in the group in which Gamma Knife administration is combined with bevacizumab use, as we had proposed mechanistically above. Patient age and number of surgical resections had significant effects on disease progression and patient death on univariate analysis, but improvements in post-recurrence PFS and OS attributed to differences in treatment were demonstrably robust on multivariate analysis when these differences in patient demographics and clinical variables were controlled between groups. Tumor volumes at the post-surgical, recurrence, and first post-treatment timepoints were also found to be significantly lower for the combination treatment group compared to the monotherapy groups on aggregate analysis. These findings reflect previous studies showing that control of tumor size does have an effect on the overall patient survival, though, ultimately, these initially observed differences lose significance over time (at Second Post-Treatment, Third Post-Treatment, and Last Recorded Tumor Volumes), suggesting that bevacizumab administration following Gamma Knife treatment has a more prominent role in sustaining long-term survival than short-term benefits gained from reresection.

Limitations of our study include a high proportion of excluded patients and its retrospective nature potentially leading to selection bias, in addition to possible confounds related to volumetric-based data collection. Contrast enhancement has been known to be influenced by factors such as corticosteroid use, postsurgical inflammation, and radiation necrosis [20], and tumor volume measurements are also complicated by operator variability, difficulty including nonenhancing portions of the tumor, and enhancement of the surgical resection cavity [38]. Decreases in tumor enhancement observed following initiation of bevacizumab therapy may be caused by improved control of peritumoral edema, rather than reductions in the actual size of the tumor (referred to as “pseudoresponse”) [39]. Temporal bias regarding the timing of Gamma Knife before or after starting bevacizumab may also confound our calculations of changes in tumor volume, as bevacizumab use may have an effect on improving Gamma Knife planning by reducing nontumor-related enhancement. An analysis of the timing of Gamma Knife and bevacizumab administration and its effects on survival outcomes may require a larger study to fully articulate their benefits. Conversely, Bevacizumab may also make it more difficult to visualize previously enhancing as well as nonenhancing lesions and thereby confound determinations of PFS predicated on observing tumor progression on MRI. However, these radiological considerations would not impact OS, which would be enough to demonstrate a therapeutic advantage to using bevacizumab and SRS when compared with other regimens reported in the literature. Future studies may benefit from the inclusion of serial neurocognitive assessments in order to follow trends in patient functional status over time and provide a better measurement of PFS.

Our results demonstrate that the use of both bevacizumab and Gamma Knife SRS may improve survival for patients with first-recurrence GBM. They further suggest that combined, multimodal approaches are required for a disease as complex as GBM, and it is the hope of the authors that our study contributes to the further development of such treatment regimens in the future.

## Conclusions

In this retrospective study, the combined use of bevacizumab and Gamma Knife in patients with recurrent glioblastoma improved progression-free and overall survival compared to patients who received only bevacizumab or Gamma Knife as monotherapy. Our findings support previous studies in the literature which suggested that a combined regimen of an antiangiogenic agent with stereotactic radiosurgery can improve survival in patients with recurrent disease, although a prospective randomized study would be required in the future to address this question more definitively.

### Supplementary Information

Below is the link to the electronic supplementary material.

Supplementary file1 (DOCX 14 KB)

## Data Availability

Data supporting the findings of this study will be made available by the corresponding author upon request.

## List of Abbreviations

ANOVA: Analysis of variance
BEV: Bevacizumab
CNS: Central nervous system
GBM: Glioblastoma
GK: Gamma Knife
Gy: Gray
HIF: Hypoxia-inducible factor
IDH: Isocitrate dehydrogenase
IQR: Interquartile range
IRB: Institutional review board
KPS: Karnofsky performance scale
MGMT: O-6 methylguanine-DNA methyltransferase
MRI: Magnetic resonance imaging
OS: Overall survival
PFS: Progression-free survival
SD: Standard deviation
SRS: Stereotactic radiosurgery
VEGF: Vascular endothelial growth factor
WT: Wild-type
