# Supplementary material for: Bevacizumab and gamma knife radiosurgery for first-recurrence glioblastoma
Source: J Neurooncol. 2024 Jan 4;166(1):89–98. doi: 10.1007/s11060-023-04524-y (PMC10824796; doi:10.1007/s11060-023-04524-y)
Supplement: Supplementary file 1 — Supplementary file1 (DOCX 14 KB) [file 11060_2023_4524_MOESM1_ESM.docx]

**Supplemental Table 1: Gamma Knife Fractions and Doses**

| Fractions | n | Prescription Dose (Gy) | | | | | Maximum Dose (Gy) | | | Prescription Isodose (%) | | |
| --- | --- | --- | --- | --- | --- | --- | --- | --- | --- | --- | --- | --- |
|  |  | | **Mean** | **Min** | | **Max** | **Mean** | **Min** | **Max** | **Mean** | **Min** | **Max** |
| 1 | 82 | | 15 | 4 | 20 | | 26.6 | 4.6 | 42.5 | 57 | 40 | 95 |
| 2 | 2 | | 10 | 10 | 10 | | 14.2 | 12.4 | 16.1 | 72 | 62 | 81 |
| 3 | 21 | | 24 | 15 | 27 | | 44.3 | 27.3 | 60.0 | 55 | 45 | 85 |
| 4 | 9 | | 20 | 20 | 20 | | 34.4 | 20.6 | 40.0 | 61 | 50 | 97 |
| 5 | 22 | | 25 | 25 | 25 | | 47.2 | 26.3 | 55.6 | 55 | 45 | 95 |
|  |  | |  |  |  | |  |  |  |  |  |  |

*Gamma Knife radiation doses and prescription isodose percentages by number of fractions administered to each lesion. Mean, minimum, and maximum dosages (in Gy) are specified for the prescribed dose and the maximum dose given.*

*Gy, Gray*
